# Association between beat-to-beat blood pressure variability and vascular elasticity in normal young adults during the cold pressor test

**DOI:** 10.1097/MD.0000000000006000

**Published:** 2017-02-24

**Authors:** Yufa Xia, Dan Wu, Zhifan Gao, Xin Liu, Qian Chen, Lijie Ren, Wanqing Wu

**Affiliations:** a Research Center for Biomedical Information Technology, Shenzhen Institute of Advance Technology, Chinese Academic of Science; b Shenzhen Children Hospital; c Department of Neurology, Shenzhen Second People's Hospital, Futian District; d Shenzhen College of Advanced Technology, University of Chinese Academy of Sciences,Shenzhen University Town, Shenzhen, China.

**Keywords:** blood pressure variability, total arterial compliance, vascular elasticity

## Abstract

The beat-to-beat blood pressure (BP) monitoring parameters, such as average beat-to-beat BP, BP variability (BPV), could have an influence on the vascular elasticity. This study hypothesized that the elevated beat-to-beat BPV could evoke the reduction of the vascular elasticity independent of BP levels. We measured the beat-to-beat BP recordings and total arterial compliance (TAC), which was used to assess the vascular elasticity, in 80 young healthy adults during the cold pressor test (CPT). The CPT included 3 phases: baseline phase, cold stimulus phase, recovery phase. Six parameters were used to estimate BPV. In bivariate correlation analysis, TAC showed a significant correlation with systolic BP (SBP) and diastolic BP (DBP) in the cold stimulus phase; and 4 indices of SBP variability (SBPV) were associated with TAC (*r* = 0.271∼0.331, *P* ≤ 0.015) in the recovery phase; similarly, 2 indices of DBP variability (DBPV) were also correlated with TAC (*r* = 0.221∼0.285, *P* ≤ 0.048) in the recovery phase. In multivariate regression analysis, DBPV (*β* = 0.229, *P* = 0.001) was a determinant of TAC independent of average DBP, sex, and weight. In addition, both beat-to-beat BP and BPV values increased in the cold stimulus phase (*P* < 0.01); whereas, the TAC decreased in the cold stimulus phase (*P* < 0.01). In conclusion, these data suggest that the beat-to-beat DBPV shows an independent association with the vascular elasticity in young normal adults during the CPT.

## 1 Introduction

According to the report from the World Health Organization, the cardiovascular disease (CVD) remained the number one cause of death globally.^[1]^ Some studies had demonstrated that aging played an important role in the progress of CVD,^[2–4]^ and the early vascular aging reflected the progress of aging on vascular tree and arterial function.^[5]^ The early vascular aging could cause the structural and functional modifications in the vasculature, accompanied by the decreased arterial elasticity and elevated arterial stiffness.^[6,7]^

Arterial elasticity could be assessed by total arterial compliance (TAC). TAC was an assessment of the stiffness of the arteries in the entire arterial tree,^[8]^ and it reflected the elasticity of the vascular system.^[9,10]^ Some studies had demonstrated that TAC played a critical role in predicting CVD.^[9–12]^ Moreover, previous studies had also manifested that TAC was an important determinant of arterial load, left ventricle (LV) afterload, and pulse wave velocities (PWV).^[13,14]^ TAC might be influenced by the blood pressure (BP) parameters, such as BP and BP variability (BPV). Recently, some studies investigated the relation between short-term BPV parameters, which included the 24-hour BPV and beat-to-beat BPV, and vascular properties.^[7,15–19]^ And they found that the increased 24-hour BPV had adverse influences on the vascular properties,^[7,15–18]^ while the relationship between vascular properties and beat-to-beat BPV was non-significant.^[19]^

Beat-to-beat BP measurement was increasingly used both in the research field and clinical research, as it could reflect more information than 24-hour BP and office BP.^[20]^ And beat-to-beat BP recordings were considered as the optimal method to obtain the short-term BPV.^[19,20]^ Moreover, beat-to-beat BP parameters had also been proved to associate with CVD and target organ damage.^[19,21]^ Accurate beat-to-beat BP used to obtain from invasive method^[22]^; the non-invasive continuous BP monitoring in the finger arterial was an excellent innovation in continuous BP measurement.

In this study, we hypothesized that the beat-to-beat BP monitoring parameters, such as systolic BP (SBP), diastolic BP (DBP), SBP variability (SBPV), and DBP variability (DBPV), could have influences on the vascular elasticity, assessed by TAC. We measured beat-to-beat BP values, TAC, electrocardiograph (ECG), and demographics in a normal young population. As the TAC could be changed in the cold pressor test (CPT),^[23]^ which had been used as a stress to estimate the cardiac autonomic function, left ventricular function, cardiovascular reactivity, and sympathetic activity,^[23,24]^ we used CPT in this study. Our aim was to investigate the relationship between vascular elasticity and beat-to-beat BPV in normal young adults during the CPT.

## 2 Materials and methods

### 2.1 Study population

The study population consisted of 80 healthy, normal young adults (51 men, 63.75%, age range 21–33 years). All the subjects met the following criteria: (a) no history of hypertension; (b) no history of diabetes mellitus, cardiopathy, or other chronic diseases; (c) no pharmacological treatment; (d) no history of drug abuse. Body weight and height were measured with the subjects without shoes and in light clothing. Body mass index (BMI) was estimated by the ratio of weight (kg) to the height square (m^2^). All the subjects were asked to refrain from caffeine, cigarette, alcohol, or severe physical activities for 3 to 4 hours prior to the experiment. They were informed of the discomfort or pain elicited by the CPT before the experiment. All subjects volunteered to participate in this study, and the written informed consent forms were obtained from all of the subjects. The study was approved by the Institutional Review Board of Shenzhen Institutes of Advanced Technology.

### 2.2 Experimental design

**Figure 1:**
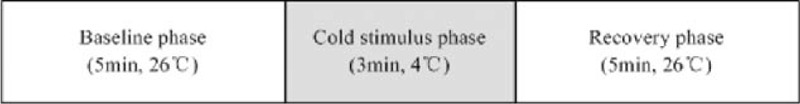
The 3 phases of the cold pressor test.

The experiment took place in a quiet temperature-controlled laboratory, and the ambient temperature was kept 26 °C. In the experiment, the subjects lay in the supine position on a single bed quietly throughout the experimental procedure. The procedure of the CPT experiment lasted 13 minutes and included 3 phases (showed in Fig. 1): the baseline phase (5 minutes), the cold stimulus phase (3 minutes), and the recovery phase (5 minutes). In the cold stimulus phase, the subject immersed his/her left hand to the wrist into a mixture of ice and water (4 °C) for 3 minutes. During the entire experimental procedure, cardiovascular signals were collected by Finometer MIDI (Model II, Finapres Medical Systems B.V., Amsterdam, The Netherlands); and the cardiovascular signals mainly included SBP, DBP, stoke volume, and electrocardiograph (ECG). Then these cardiovascular signals were stored in a personal computer by the BeatScope Easy software (Finapres Medical Systems B.V., Amsterdam, The Netherlands). The BP was measured by the volume-clamp method in the Finometer MIDI^[25]^; in the volume-clamp method, a cuff wrapped around the finger to keep the arterial diameter constant, and the changes in the arterial diameter were detected by photo-plethysmography^[25]^; physiocal algorithm was used to maintain the correct arterial diameter.^[26]^ The stroke volume was measured by Modelflow method^[25]^; the Modelflow method was utilized to calculate an aortic flow waveform from the finger pressure by simulating a non-linear three-element model of aortic input impedance.^[25]^

### 2.3 TAC assessment

There was no “gold standard” to assess the TAC. Chemla et al^[13]^ proposed the ratio of stroke volume to pulse pressure (SV/PP) to measure TAC. The pulse pressure represented the difference between SBP and DBP readings. The SV/PP had been proven reliable to assess the TAC.^[11]^ Therefore, we used SV/PP to estimate TAC in this study.

### 2.4 BPV assessment

Usually, BPV was evaluated by the standard deviation (SD) and coefficient of variation (CV) of BP measurements.^[27]^ However, the assessment by SD might be unreliable in multivariate module,^[28,29]^ because SD only reflected the global fluctuation of BP measurements around the mean value and did not take the time sequence of measurements into account. CV was derived from SD and mean BP, and it might be correlated with mean BP. To overcome these disadvantages of the commonly used SD and CV, average real variability (ARV), variation independent of mean (VIM), residual standard deviation (RSD), and successive variation (SV) were established to estimate the prognostic significance of BPV.^[28–31]^ ARV took the time sequence of individual BP measurements into account, and it could also estimate the trend of BPV without making any assumptions regarding the shape of the relationship between BP values and time.^[28]^ VIM was a transformation of SD that was uncorrelated with mean levels.^[29]^ When VIM was used as an index of BPV, it could eliminate the effects of mean BP levels. RSD was defined as the square root of the residual mean square after fitting a linear regression to BP against time^[30]^; and it was more suitable than SD to estimated BPV when the BP fluctuation had an underlying trend over time, especially the underlying trend was approximately linear. SV was defined as the square root of the average squared difference between successive BP measurements; and it also addressed the time sequence of measurements.^[31]^ In this study, SD, CV, VIM, ARV, SV, and RSD were used to assess BPV. Because the BPV included SBPV and DBPV, we used these indices to quantify SBPV and DBPV, respectively. These parameters were calculated using the following formulas: 
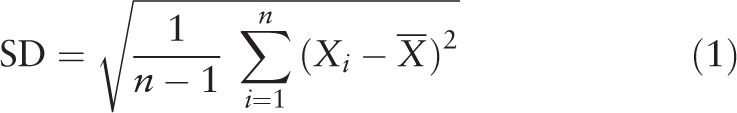
 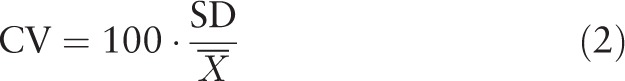 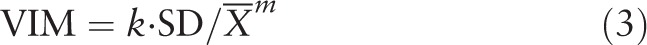 
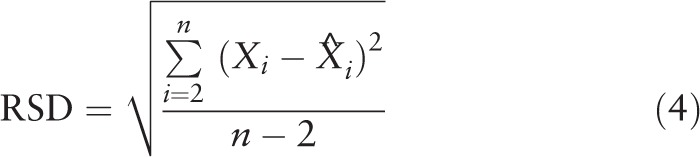
 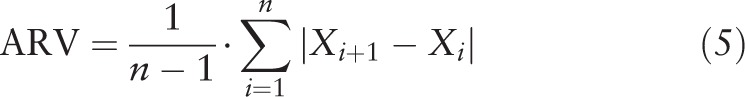 
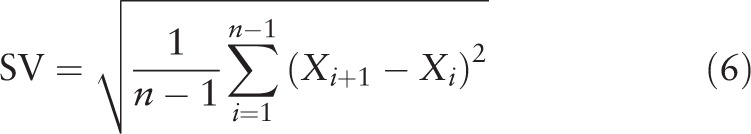


Where *X*_1_, *X*_2_, …, *X*_*n*_ represented a set of BP measurement values;  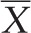 was the mean value of the set of BP measurement values;  
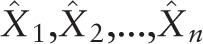
 were the fitted values from a linear regression of BP against time; *k* and *m* were obtained from a fitting curve of the form *y* = *kx*^*p*^ through a plot of SD BP (*y*-axis) against mean BP (*x*-axis).

### 2.5 HR variability assessment

HR variability (HRV) had become a significant method to estimate the cardiovascular autonomic regulation.^[32]^ We assessed the HRV from ECG readings. In time-domain, the SD of R-R intervals (SDNN) were used to estimate the HRV^[32]^; in frequency-domain, spectrum estimate was calculated from the RR interval^[33]^; we main calculated low frequency (LF, 0.04–0.15 Hz), high frequency (HF, 0.15–0.4 Hz), and the ratio between LF and HF (LF/HF).

### 2.6 Statistical analysis

The Statistical Package for the Social Sciences (SPSS) 19.0 (SPSS Inc., Chicago, IL) was used for statistical analysis. Descriptive statistics were presented as mean ± SD. Repeated-measures analysis of variance (ANOVA) was used to test the differences of parameters among the 3 phases of CPT. Pearson correlation coefficients were used to investigate the bivariate associations between TAC and examined variables. Stepwise multivariate linear regression analysis (stepwise criteria: probability of F-to-enter ≤0.050, probability of F-to-remove ≥0.100) was used to elucidate the independent determinants of TAC. *P* < 0.05 was considered statistically significant.

## 3 Results

### 3.1 Demographic and clinical characteristics data

Demographic data, BP and HR values of the population were listed in Table 1. The 80 subjects included 51 men (63.75%). Their age (mean ± SD) was 25.2 ± 2.4 years, and the BMI was 21.2 ± 2.1 kg/m^2^. Their SBP was 111.7 ± 9.7 mmHg, and their DBP was 62.9 ± 6.6 mmHg. Their HR was 67.1 ± 10.3 beats/min. None of them was a smoker.

**Table 1:**
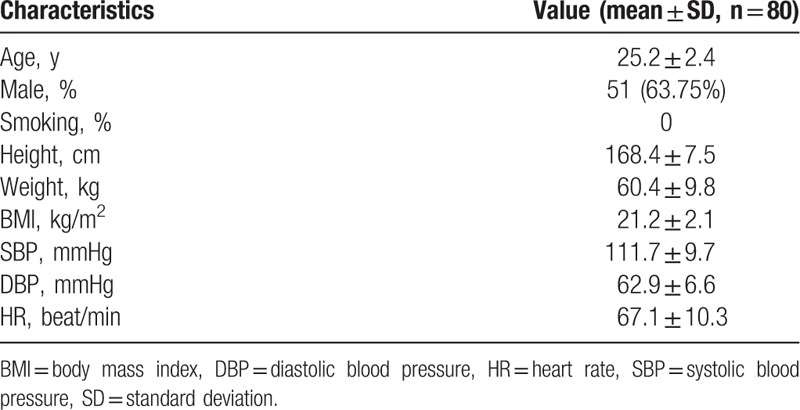
Clinical characteristics of the study subjects.

### 3.2 Repeated-measures analysis of variance analysis

The beat-to-beat BP, BPV, HR, HRV, and TAC values in the 3 phases of the CPT were presented in Table 2. The SBP and DBP values in the cold stimulus phase were significantly larger than the values both in the baseline phase and the recovery phase (*P* < 0.001). Moreover, all of the 6 indices of SBPV and DBPV in the cold stimulus phase were also remarkably larger than the values in the baseline phase and the recovery phase (*P* < 0.001). Similarly, HR values in the cold stimulus phase were larger than the values in the baseline phase and the recovery phase (*P* < 0.001) significantly. Moreover, SD of HR, SDNN, LF, and LF/HF in the cold stimulus phase were also larger than the values in the baseline and recovery phases (*P* < 0.001, *P* = 0.001, *P* = 0.034, *P* = 0.008, respectively). Whereas, the RR interval and HF in the cold stimulus phase were smaller than the values in the baseline and recovery phases (*P* = 0.034, *P* = 0.001, respectively). Similarly, TAC values in the cold stimulus phase were smaller than the values in the baseline phase and the recovery phase (*P* < 0.001).

**Table 2:**
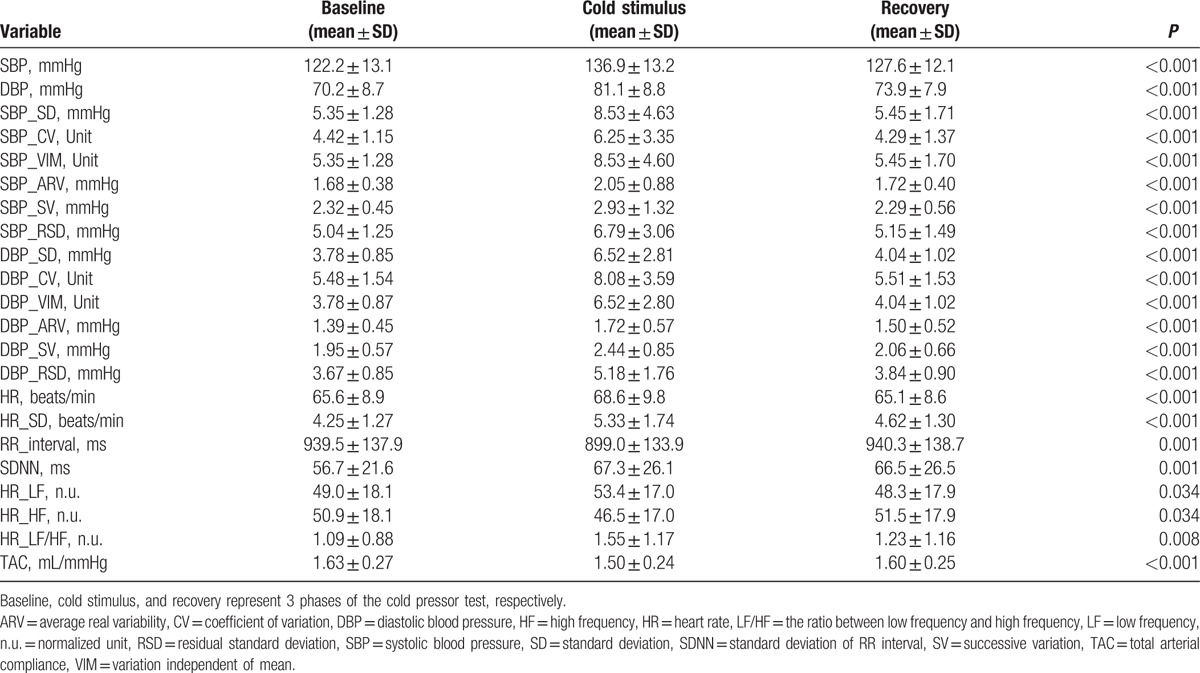
The beat-to-beat blood pressure parameters, heart rate parameters, and total arterial compliance in 3 phases of cold pressor test.

### 3.3 Bivariate correlation analysis

Pearson correlations of TAC with the beat-to-beat BP, BPV, HR, HRV, and demographic data in the 3 phases of CPT were shown in Table 3. TAC showed a significant correlation with sex, height, weight, BMI in all of the 3 phases of CPT. In the baseline phase, LF and HF of HR showed a significant correlation with TAC (*r* = 0.308, *P* = 0.010; *r* = −0.306, *P* = 0.010, respectively). In the cold stimulus phase, both SBP and DBP were related to TAC remarkably (*r* = 0.291, *P* = 0.009; *r* = 0.339, *P* = 0.002; respectively). In the recovery phase, neither SBP nor DBP was correlated with TAC remarkably (*P* ≥ 0.054); whereas, SD, CV, VIM, and RSD of SBP were associated with TAC significantly (*r* = 0.271, *P* = 0.015; *r* = 0.331, *P* = 0.003; *r* = 0.285, *P* = 0.010; *r* = 0.282, *P* = 0.011; respectively); CV and VIM of DBP were also correlated with TAC remarkably (*r* = 0.285, *P* = 0.010; *r* = 0.221, *P* = 0.048; respectively). Furthermore, SDNN, LF, and HF of HR also showed an important correlation with TAC (*r* = 0.234, *P* = 0.044; *r* = 0.257, *P* = 0.032; *r* = 0.256, *P* = 0.032; respectively) in the recovery phase.

**Table 3:**
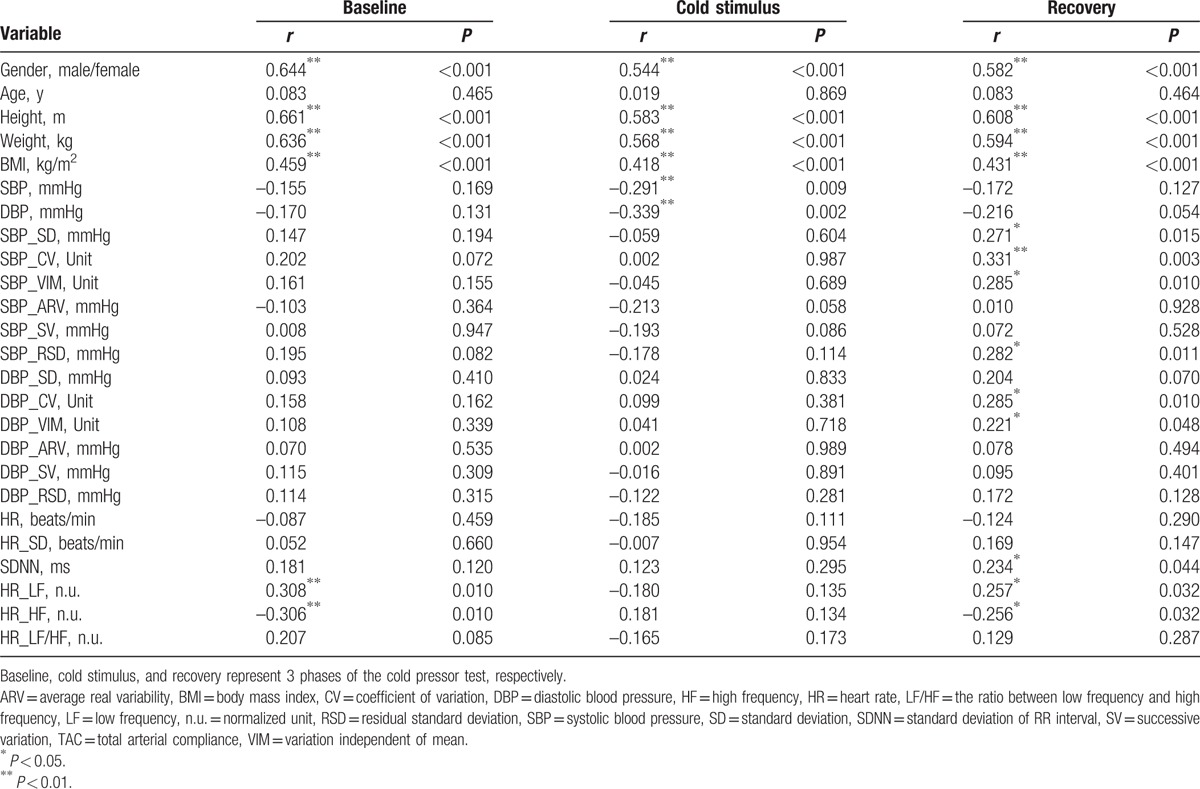
Pearson correlation of total arterial compliance with demographic data, heart rate, beat-to-beat blood pressure parameters.

### 3.4 Stepwise multivariate linear regression analysis

**Figure 2:**
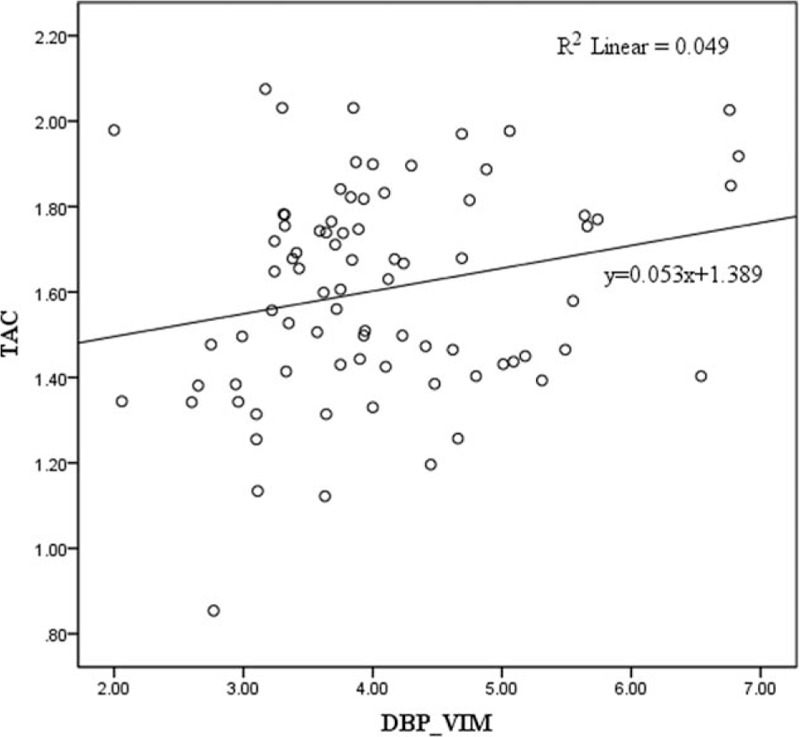
The linear relationship between TAC and VIM of DBP in the recovery phase of CPT. CPT = cold pressor test, DBP = diastolic blood pressure, TAC = total arterial compliance, VIM = variation independent of mean.

Tables 4 and 5 showed the stepwise multivariate linear regression analysis of the TAC with the demographics, beat-to-beat BP parameters and HR parameters in the cold stimulus phase and the recovery phase, respectively. The independent relationship between TAC and BPV was demonstrated in a stepwise multivariate linear regression model. VIM of DBP was associated with TAC independent of DBP, sex and weight in the recovery phase of the CPT. Figure 2 showed the linear relationship between TAC and VIM of DBP in the recovery phase of CPT. Whereas neither SBP nor SBPV showed significant correlation with TAC in cold stimulus phase or recovery phase.

**Table 4:**
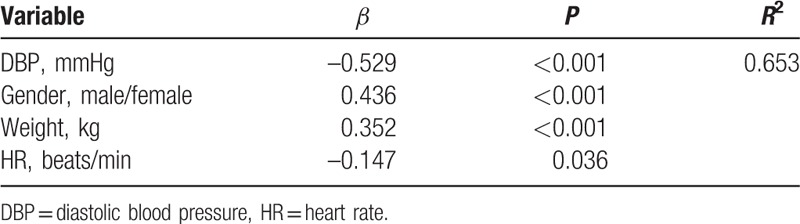
Independent predictors of total arterial compliance in multivariate linear regression analysis during cold stimulus phase of cold pressor test.

**Table 5:**
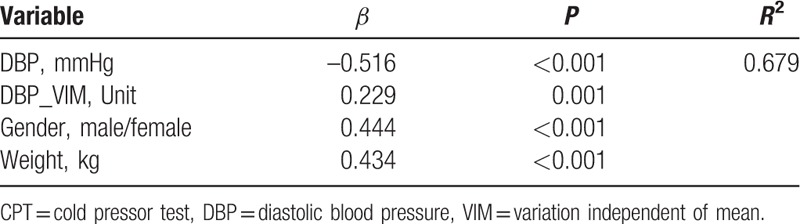
Independent predictors of total arterial compliance in multivariate linear regression analysis during recovery phase of cold pressor test.

## 4 Discussion

In this study, our results offer explicit evidence of the significant correlations between vascular elasticity, as estimated by TAC, and different measures of beat-to-beat SBPV and DBPV in young normal adults during the CPT. It implies that increased beat-to-beat BPV has an important influence on structural and functional vascular properties, and increases the progress of the early vascular aging in young normal adults independent of mean BP levels.

In addition, our study also has some other merits. Firstly, the association of TAC with beat-to-beat BP has been confirmed in the cold stimulus phase of the CPT. Secondly, the augmentation of mean beat-to-beat BP and BPV values is confirmed in the cold stimulus phase of the CPT. Thirdly, HR and HRV in time-domain also increase in the cold stimulus phase; the LF and LF/HF in frequency increase, but the HF decrease in the cold stimulus phase. Fourthly, in the bivariate correlation analysis, LF and HF component of HRV had a significant correlation with TAC in baseline and recovery phases. Lastly, the reduction of the TAC is found in the cold stimulus phase of the CPT.

These experimental results may have some potential clinical implications. Even in normal young adults, the increase of BPV has an adverse influence of the vascular elasticity independent of BP. So not only keeping the BP in a normal range but also decreasing the BPV may be helpful to prevent the early cardiovascular diseases and complications. Furthermore, the increase of BPV may predict the early vascular lesions, and it can be as a mark of premature cardiovascular diseases. The decreased BPV may be also advantageous for the treatment of cardiovascular diseases.

There is a physiological inference to explicate our results. The beat-to-beat BPV mainly reflects the effects of the central sympathetic drive, arterial or cardiopulmonary reflex, humoral, rheological, behavioral, and emotional factors.^[20]^ When the vascular elasticity reduces, it can be associated with the change of the BPV. Moreover, previous studies have explored the relationship between vascular properties and short-term BPV both in healthy population^[7]^ and in patient population.^[15–19]^ In these studies, PWV was used to assess the arterial stiffness, and intima-media thickness (IMT) was a surrogate marker for atherosclerosis. Kotsis et al^[7]^ studied 115 young healthy volunteers; and they manifested that increased 24-hour SBPV associated with arterial stiffness in young healthy population. Schillaci et al^[15]^ explored the relationship between 24-hour BPV and aortic stiffness in hypertensive population, and found that the association between ARV and PWV was closer than other indices; and they thought that ARV focused on the very short-term BPV between successive readings. Stabouli et al^[16]^ studied 138 children and adolescents patients; and they attested that weighted 24-hour and daytime SBPV associated with arterial stiffness in children and adolescents. Tatasciore et al^[17]^ studied the correlation between 24-hour BPV and IMT in 180 untreated hypertensive subjects; and they found that awake SBPV was a better correlate of IMT than 24 hour SBPV. Mancia et al^[18]^ explored 1663 hypertensive patients; they also manifested that 24-h SBPV was related to IMT. Furthermore, Wei et al^[19]^ investigated the relationship between arterial stiffness and BPV over different periods in 256 untreated patients; they used ARV, VIM, and maximum–minimum difference (MMD) to assess the BPV; and they found that the PWV increased with beat-to-beat SBP, but did not increase with beat-to-beat SBPV; moreover, they also found that the PWV increased with 24-hour SBP and VIM of 24-hour SBP. In stepwise multivariate linear regression analysis, our results showed that VIM of DBP was an independent determinant of the vascular elasticity, but the correlation of TAC with SBPV was not significant. The different methods to estimate the BPV and vascular properties might cause the difference.

Interestingly, in the present study, some indices of beat-to-beat BPV correlated with the TAC in the recovery phase of the CPT; whereas, no indices of beat-to-beat BPV associated with TAC in the baseline phase and cold stimulus phase in young normal adults. The results showed that the BPV increased from the baseline phase to the cold stimulus phase, while it decreased from the cold stimulus phase to the recovery phase. However, the change of TAC was contrary to the change of BPV during the CPT. It suggests that the reduced BPV may have an influence on the increase in TAC.

In addition, our results also showed that beat-to-beat BP levels and BPV values increased in the cold stimulus phase of the CPT. The increased beat-to-beat BP levels and BPV values in the cold stimulus phase were due to the increased sympathetic nervous system (SNS) activity. The analysis of HRV is a reliable method to assess the autonomic nervous system.^[32]^ The LF component (0.04–0.15 Hz) is regarded as a marker of SNS, and the HF component (0.15–0.4 Hz) mainly reflects the parasympathetic nerve system (PNS) activity^[33]^; the LF/HF is comprehensive measure of autonomic balance.^[32]^ Our results showed that the LF and LF/HF increased, while the HF decreased in the cold stimulus phase. It suggested that the cold stimulus increased the SNS activity and reduced the PNS activity. The SNS activity caused the constriction of resistance vessels and resulted in the increase of the BP level and BPV value.^[24]^ Weise et al^[34]^ also investigated the influence of the CPT on short-term fluctuations of finger arterial BP; they tested 10 healthy subjects’ BP, and manifested that the cold stimulus induced significant increases in SBP and DBP; whereas, the changes of SBPV and DBPV, assessed by SD, were not significant. The small size of the subjects and the BPV assessed only by SD might contribute to the changes of BPV non-significant in their study.

Furthermore, the reduction of the TAC during the CPT in young normal adults may result from the increased SNS activity and the elevated BP. Some evidences show that the TAC decreases with aging, BP, CVD, and SNS activity.^[12,35]^ The changes of HRV in frequency domain during the CPT have confirmed that the cold stimulus evoked the increase in SNS activity. Increased SNS activity led to the reduction of the TAC. Boutouyrie et al^[35]^ used a non-invasive method to investigate the impact of SNS activity on arterial compliance in normal subject; and they also demonstrated that SNS activity could induce a reduction of TAC. Furthermore, our results exhibited that the TAC had significant correlation with LF and HF component during the baseline and recovery phase of CPT, while the correlation of TAC with LF and HF component was not significant in cold stimulus phase. The reduction of TAC might lead to the correlation of TAC with LF and HF component became non-significant in cold stimulus phase.

Notwithstanding the novelty of our results, we should interpret the limitations of our study. On the one hand, the current study was a cross-sectional study. Given the nature of cross-sectional design, we could not elucidate whether the elevated BPV induced the reduction of the TAC or versa. Larger longitudinal studies are needed to estimate the prognostic implications of beat-to-beat BPV on TAC in young normal adults. On the other hand, the sample size of the present study was relatively small. The relatively small sample size may have some influences on the results.

## 5 Conclusion

In conclusion, the results of this study indicate that the beat-to-beat BPV associates with the TAC in the recovery phase of the CPT. As TAC reflects the elasticity of the vascular system, it implies that the increased beat-to-beat BPV has an impact on vascular elasticity, and elevates the progress of the early vascular aging in normal young adults. In addition, we have also confirmed that the cold stimulus increased SNS activity and evoked the increase in BP levels and variability, but reduced the TAC. Furthermore, more clinical experiments are needed to confirm the correlations between beat-to-beat BPV and vascular elasticity not only in healthy population but also in hypertensive population.

## Acknowledgments

The authors gratefully acknowledge the volunteers who participated in our study.
